# Supplementary material for: Increased Cerebral Level of P2X7R in a Tauopathy Mouse Model by PET Using [18F]GSK1482160
Source: ACS Chem Neurosci. 2024 May 22;15(11):2112–20. doi: 10.1021/acschemneuro.4c00067 (PMC11157487; doi:10.1021/acschemneuro.4c00067)
Supplement: Supplementary file 1 — cn4c00067_si_001.pdf [file cn4c00067_si_001.pdf]

Supporting information

Increased cerebral level of P2X7R in a tauopathy mouse model by PET using [^{18}F]GSK1482160

Yanyan Kong[1], Lei Cao[1,2], Jiao Wang[3], Junyi Zhuang[3], Yongshan Liu[4], Lei Bi[4], Yifan Qiu[4], Yuyi Hou[4], Qi Huang[1], Fang Xie[1], Yunhao Yang[1], Kuangyu Shi[5], Axel Rominger[5], Yihui Guan[1], Hongjun Jin[4]*, Ruiqing Ni[2,5,6]*

[1]PET Center, Huashan Hospital, Fudan University, Shanghai, China

[2]Institute for Regenerative Medicine, University of Zurich, Zurich, Switzerland

[3]Lab of Molecular Neural Biology, School of Life Sciences, Shanghai University, Shanghai, China

[4]Guangdong Provincial Engineering Research Center of Molecular Imaging, the Fifth Affiliated Hospital, Sun Yat-Sen University, Zhuhai, 519000, Guangdong Province, China

[5]Department of Nuclear Medicine, University Hospital, Inselspital Bern, Bern, Switzerland

[6]Institute for Biomedical Engineering, University of Zurich & ETH Zurich, Zurich, Switzerland

Corresponding author:
Hongjun Jin
Email: jinhj3@mail.sysu.edu.cn
Address: 52 E, Meihua Road, Zhuhai 519000, China

Ruiqing Ni
Email: ni@biomed.ee.ethz.ch
Address: Wolfgang Pauli strasse 27, HIT E22.1 Zurich 8093 Switzerland

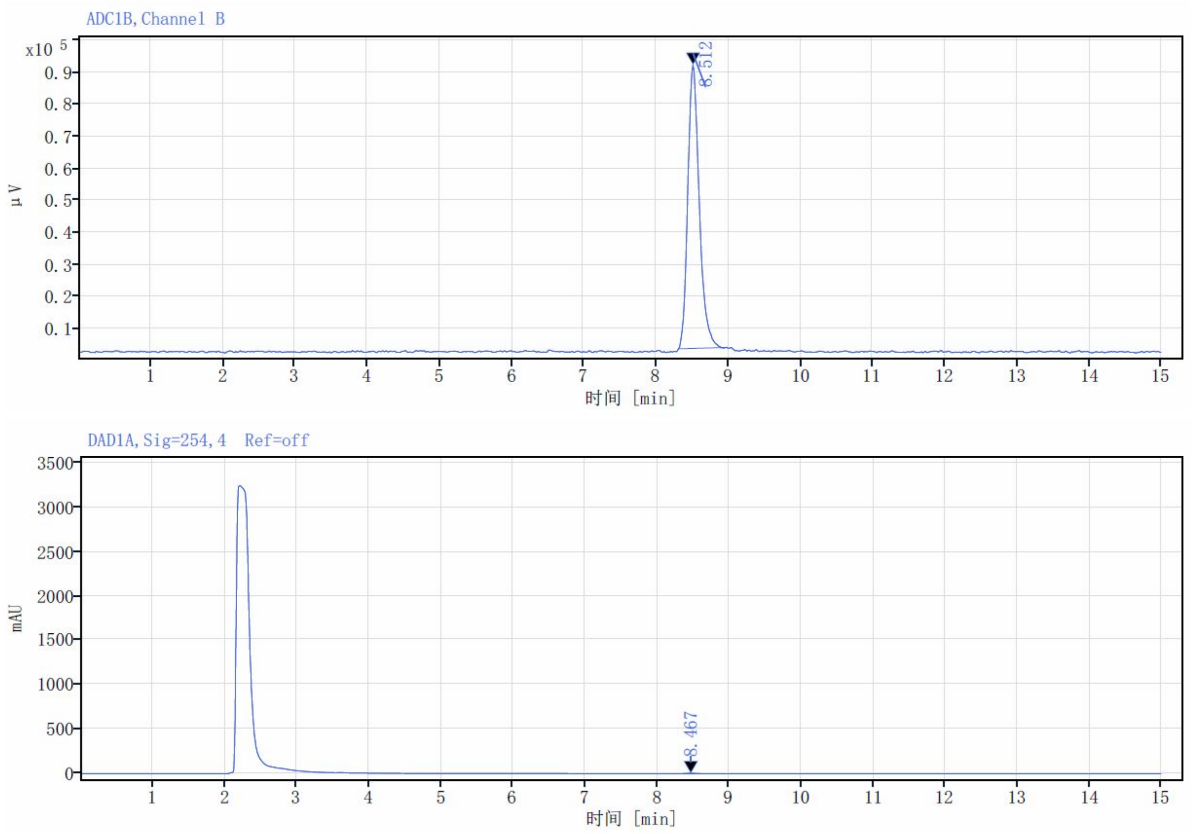

Figure S1. HPLC chromatogram of synthesized [^{18}F]GSK1482160 and the standard. (**a**) Synthesized GSK1482160 and (**b**) standard.

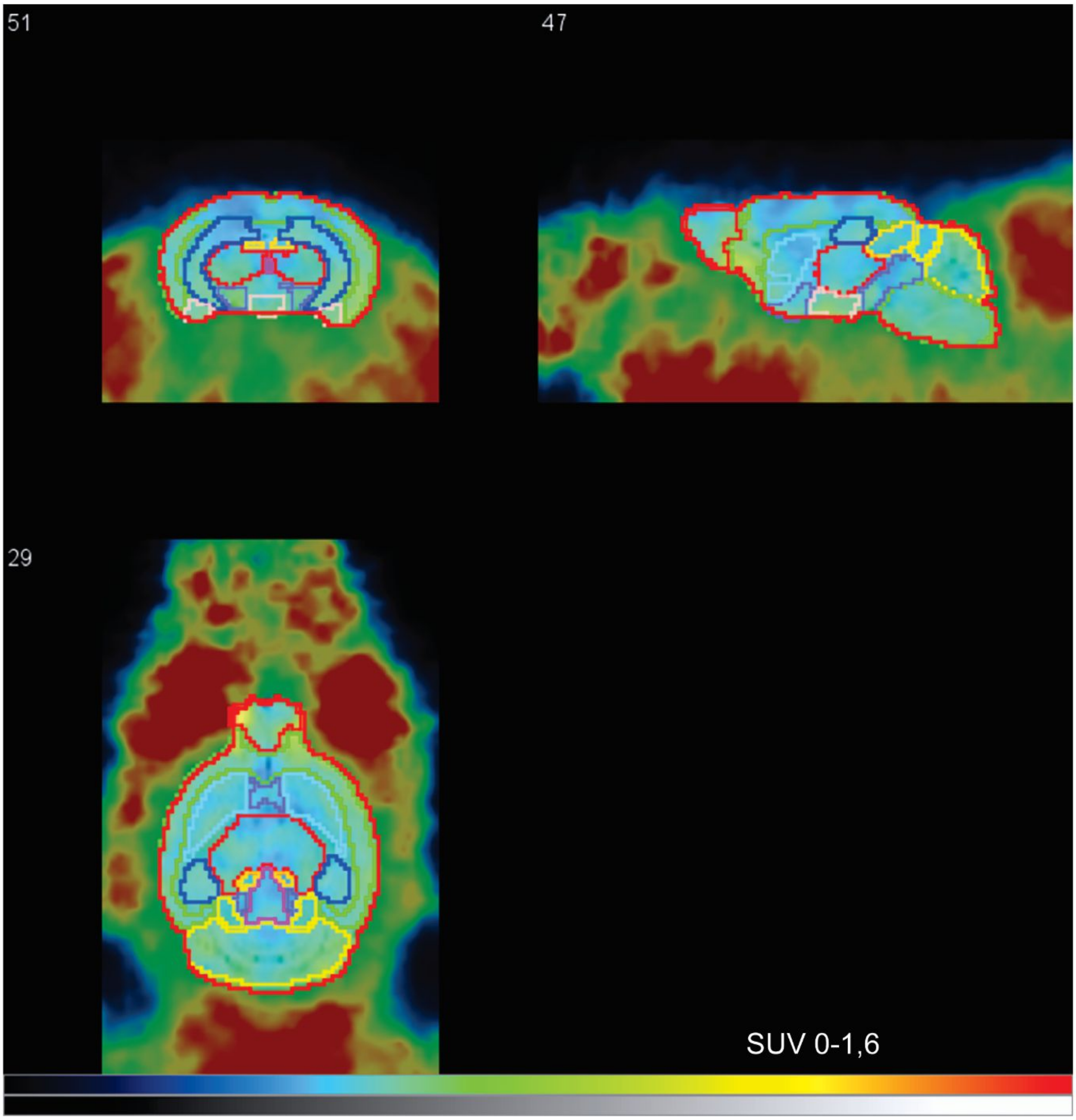

Figure S2. [^{18}F]GSK1482160 exhibited relatively lower brain uptake than outside the brain in wild-type mice (averaged 50-60 minutes post injection). The colour bar indicates SUV 0-1.6. The template used for volume-of interest analysis (overlaid on the images) was the Ma-Benveniste-Mirrione atlas.

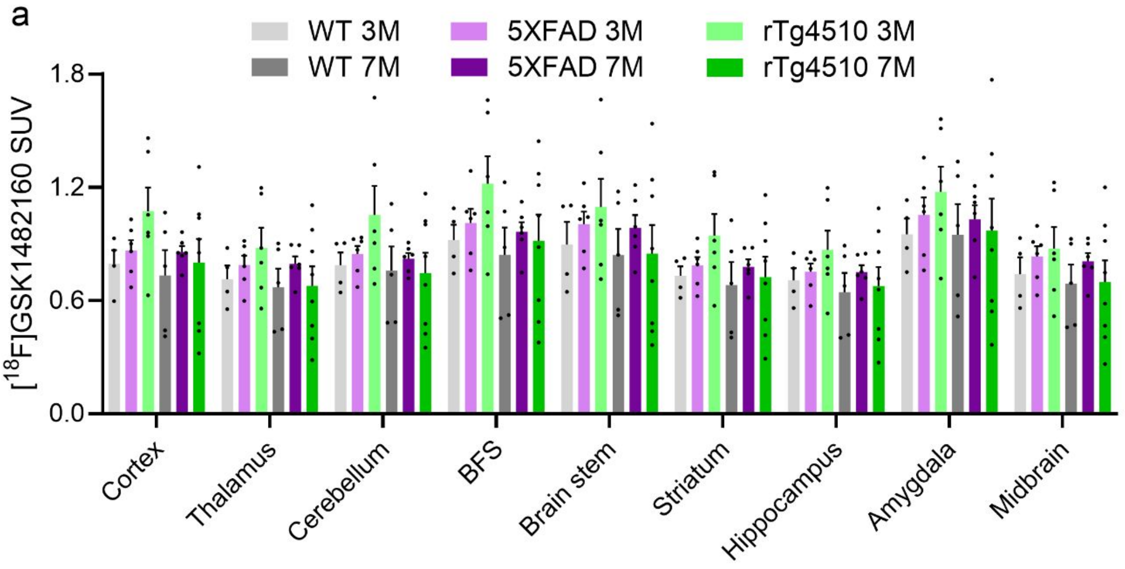

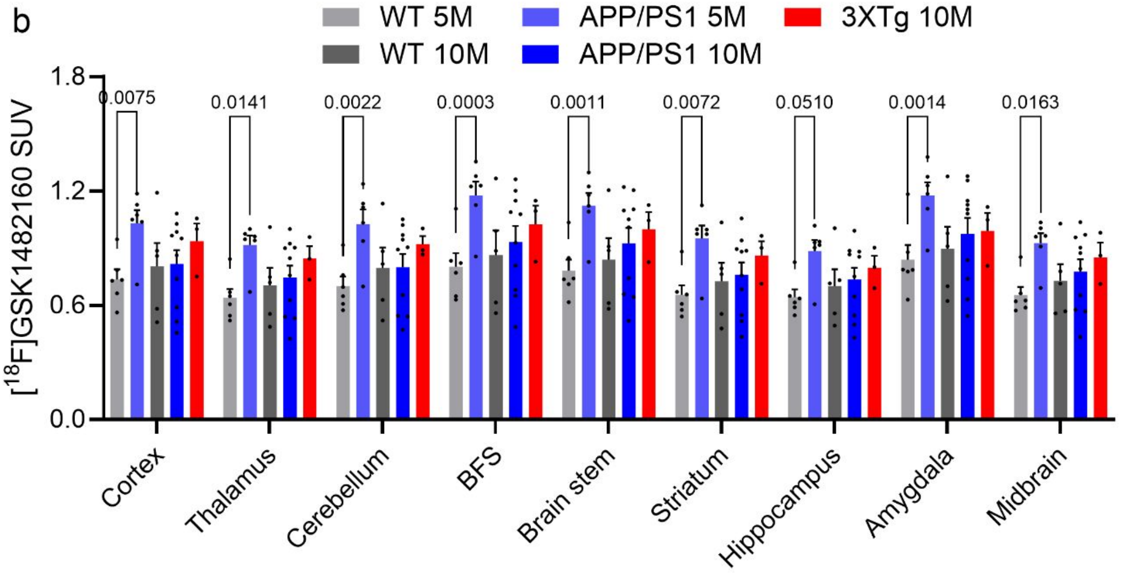

Figure S3. [^{18}F]GSK1482160 SUV in the brain of WT, rTg4510, 5×FAD, APP/PS1 and 3×Tg mice. (**a**) in the brain of 3 month old and 7 months old WT, rTg4510, and 5×FAD mice, (**b**) in the brain of 5 month old and 10 months old WT, and APP/PS1, and 10 months old 3×Tg mice. Two-way ANNOVA with Sidek'post hoc analysis. BFS, basal forebrain system.

Table S1. Antibodies and reagents used for immunofluorescence staining

Antibodies and reagent	Catalog no	Dilution	Supplier
Rat monoclonal anti-P2X7 antibody [1F11]	Ab195356	1:200	Abcam
CY3-conjugated goat anti-rat IgG	Gb21302	1:300	Servicebio
Anti-Amyloid beta 40, mouse mAb	GB121197	1:1200	Servicebio
Goat Anti-Mouse IgG H&L (Alexa Fluor® 488)	ab150113	1:500	Abcam
Anti-phospho-TAU (S202/T205), rabbit polyclonal antibody	GB113883	1:1000	Servicebio
Alexa Fluor488 goat anti-rabbit IgG	GB25303	1:400	Servicebio
CY3-conjugated goat anti-mouse IgG	GB21301	1:300	Servicebio
Bovine serum albumin	36100ES25	3%	Yeasen
TritonX-100	X10010	0.4%	Abcone
Normal goat serum (NGS)	36119ES03	5%	Yeasen
4′,6-diamidino-2-phenylindole (DAPI)	D8200	1:2000	Solarbio
Citrate buffer pH 6.0	G1202		Servicebio
Phosphate buffered saline	G0002		Servicebio
Anti-fluorescence quenching mounting media	36307ES08		Yeasen
